# Comparative study of the binding characteristics to and inhibitory potencies towards PARP and in vivo antidiabetogenic potencies of taurine, 3-aminobenzamide and nicotinamide 

**DOI:** 10.1186/1423-0127-17-S1-S16

**Published:** 2010-08-24

**Authors:** Kashyap G Pandya, Maulik R Patel, Cesar A Lau-Cam

**Affiliations:** 1 Department of Pharmaceutical Sciences, St. John’s University, College of Pharmacy and Allied Health Professions, 8000 Utopia Parkway, Jamaica, New York 11439, USA

## Abstract

**Background:**

Poly(ADP-ribose) is a NAD^+^-requiring, DNA-repairing, enzyme playing a central role in pancreatic β-cell death and in the development of endothelial dysfunction in humans and experimental animals. PARP activation is also relevant to the development of complications of diabetes. Hence, agents capable of inhibiting PARP may be useful in preventing the development of diabetes and in slowing down complications of diabetes.

**Methods:**

PARP inhibition was assessed with a colorimetric assay kit. Molecular docking studies on the active site of PARP were conducted using the crystalline structure of the enzyme available as Protein Data Bank Identification No. 1UK1. Type 2 diabetes was induced in male Sprague-Dawley rats with streptozotocin (STZ, 60 mg/kg, i.p.). The test compounds (3-aminobenzamide = 3-AB, nicotinamide = NIC, taurine = TAU) were given by the i.p. route 45 min before STZ at 2.4 mM/kg (all three compounds) or 1.2 and 3.6 mM/kg (only NIC and TAU). Blood samples were collected at 24 hr after STZ and processed for their plasma. The plasma samples were used to measure glucose, insulin, cholesterol, triglycerides, malondialdehyde, nitric oxide, and glutathione levels using reported methods.

**Results:**

3-AB, NIC and TAU were able to inhibit PARP, with the inhibitory potency order being 3-AB>NIC>>TAU. Molecular docking studies at the active site of PARP showed 3-AB and NIC to interact with the binding site for the nicotinamide moiety of NAD^+^ and TAU to interact with the binding site for the adenine moiety of NAD^+^. While STZ-induced diabetes elevated all the experimental parameters examined and lowered the insulin output, a pretreatment with 3-AB, NIC or TAU reversed these trends to a significant extent. At a dose of 2.4 mm/kg, the protective effect decreased in the approximate order 3-AB>NIC≥TAU. The attenuating actions of both NIC and TAU were dose-related except for the plasma lipids since NIC was without a significant effect at all doses tested.

**Conclusions:**

At equal molar doses, 3-AB was generally more potent than either TAU or NIC as an antidiabetogenic agent, but the differences were not as dramatic as would have been predicted from their differences in PARP inhibitory potencies. NIC and TAU demonstrated dose-related effects, which in the case of TAU were only evident at doses ≥2.4 mM/kg. The present results also suggest that in the case of NIC and TAU an increase in dose will enhance the magnitude of their attenuating actions on diabetes-related biochemical alterations to that achieved with a stronger PARP inhibitor such as 3-AB. Hence, dosing will play a critical role in clinical studies assessing the merits of NIC and TAU as diabetes-preventing agents.

## Background

Poly(ADP-ribose) polymerases (PARPs) are of group of at least 18 different cell signaling enzymes that catalyze the transfer of ADP-ribose units from NAD^+^ to a number of acceptor molecules [1]. Among PARPs, PARP-1 has attracted a great deal of attention because of its relevance to the development of type 1 diabetes and its complications. This chromatin-bound nuclear enzyme is activated upon DNA injury as a result of genotoxic stress by oxidants, oxygen-derived free radicals and nitric oxide (NO) and, upon binding to single-strand DNA breaks it recruits a ligase complex to carry out base excision repair and, at the same time, it cleaves nicotinamide adenine dinucleotide (NAD^+^) into nicotinamide and ADP-ribose residues which are covalently attached to nuclear and extranuclear (e.g. mitochondrial) proteins to form poly(ADP-ribose) [2-5]. Continuous PARP activity in the pancreas leads to large amounts of the (ADP-ribose) polymer, the depletion of NAD^+^ to nonphysiological levels, a decrease in protein synthesis, inhibition of insulin synthesis, and necrotic β-cell death [6-8]. Overactivity of PARP is also associated with ATP depletion because of increased use to regenerate NAD^+^ from nicotinamide, impaired mitochondrial function and a slow rate of glycolysis [3,5,9]. When ATP levels decrease below a critical threshold following proapoptotic insults, necrotic cell death ensues [3,10].

PARP inhibitors have the ability to prevent intracellular NAD^+^ consumption and decreases of the ATP pool, and to protect pancreatic β-cells from chemically induced necrosis but not from cytokine-mediated apoptosis, a major cause of autoimmune diabetes [11]. Furthermore, PARP inhibition has been found to not only prevent but also reverse aortic endothelial dysfunction in animals made diabetic through pharmacological intervention. At the same time, PARP inhibition was able to reverse the losses of endothelial ATP, NAD^+^ and NADPH caused by diabetes [11]. Because of the worldwide rise in the incidence of diabetes mellitus and the epidemic proportions reached by this endocrine disorder, compounds classified as PARP inhibitors are continually being tested for preventing the development of diabetes and its complications and for reversing diabetes-related organ and nerve dysfunction [11-13]. However, since complete inhibition of PARP will deprive cells from the benefits of DNA repair during periods of oxidative stress, thus contributing to cytotoxicity [14], studies on highly potent first generation PARP inhibitors like 3-aminobenzamide (3-AB) has been limited to laboratory animals. Another beneficial effect of PARP inhibitors like 3-AB, in addition to preventing pancreatic damage by a diabetogenic agent, is to preserve the ability of the pancreas to secrete insulin in response to hyperglycemia [15]. In contrast, nicotinamide (NIC), a weaker first-generation PARP inhibitor and a precursor of NAD^+^, has been the subject of intensive clinical trials around the world as a means of preventing or delaying the clinical onset of diabetes in humans [12,16-18]. In addition, this water-soluble form of vitamin B_3_ possesses other properties that may be of benefit in diabetes such as antioxidant action, modulatory role on neuronal Ca^++^ influx, inhibitory action on apoptosis [5], and ability to preserve residual β-cell function, enhance β-cell regeneration, and promote islet cell growth [18].

Among natural compounds, taurine (TAU) is among those most extensively investigated for its attenuating effects on diabetes-related alterations such as decreased insulin secretion [19,20], hyperglycemia [19,21] hyperlipidemia [21,22], lipid peroxidation (LPO) [21-23], and formation of advanced protein glycation products [23] in animal models of spontaneous and pharmacologically-induced diabetes. By analogy to 3-AB and NIC, in nonobese diabetic (NOD) mice TAU was found to promote islet cell proliferation, lower the incidence of pancreatic apoptosis, reduce islet cell insulitis and delay the onset of diabetes when consumed as a diet supplement early in life [24].

In view of the similarity of protective actions manifested by TAU, 3-AB and NIC against diabetes it appeared of interest to determine whether or not TAU is endowed with PARP-inhibiting action and, if this is the case, to what extent differences in PARP-inhibiting potency among TAU, 3-AB and NIC impact on the extent of their antidiabetic effects. To attain these goals, TAU, 3-AB and NIC were tested both in vitro for PARP-1 inhibiting power and for mode of interaction with the active site of PARP-1 in rats and in vivo, using rats made diabetic with streptozotocin, (STZ), a known generator of oxygen and nitrogen reactive species, for their counteracting actions on hypoinsulinemia, hyperglycemia, hyperlipidemia and oxidative stress when administered in equimolar doses as a pretreatment to STZ. To our knowledge these comparisons have not been previously reported.

## Methods

### Animals

All the experiments were carried on male Sprague-Dawley rats, 200-225 g in weight, purchased from Taconic Farms, Germantown, New York, USA, and housed in a temperature controlled room (21±1°C) with a 12 hr light-12 hr dark cycle. The animals were allowed free access to a standard rat chow and filtered tap water for at least 5 days. The solid food, but not the water, was removed 12 hr prior to an experiment. The experimental groups consisted of 6 animals each. The study received the approval of the Institutional Animal Care and Use Committee of St. John’s University, and the animals were cared in accordance with the guidelines established by the United States Department of Agriculture.

### Treatments and samples

Solutions of STZ, 3-AB, NIC and TAU were made in citrate buffer pH 4.6, and they were administered by the intraperitoneal (i.p.) route in a volume not exceeding 2 ml. The doses used were: STZ 60 mg/kg, 3-AB 2.4 mM/kg, TAU 1.2-3.6 mM/kg, NIC 1.2-3.6 mM/kg. 3-AB and NIC were administered 45 min before STZ. TAU was administered in divided doses, one-half at 75 min before STZ and one-half at 45 min before STZ. Control animals received only citrate buffer pH 4.6 in a volume equal to that of a treatment solution. All the animals were decapitated at 24 hr after a treatment with STZ, and their blood samples, collected into heparinized tubes, were processed for their plasma fractions.

### PARP inhibitory action

The inhibitory action of the test compounds towards PARP-1 was determined using a commercially available microplate assay kit (Universal Colorimetric PARP Assay from Trevigen, Inc., Gaithersburg, MD) and in accordance with the instructions provided by the manufacturer. Stock solutions of the various test compounds were made in dimethyl sufoxide, which were then serially diluted to the required concentration (100 µM) with distilled water. For the assay, each strip well was filled with 10 µL of the inhibitor solution, 15 μL of diluted PARP-1 enzyme (providing 0.5 Unit/well), and 25 µL of PARP Cocktail (consisting of biotinylated NAD, activated DNA in Tris-Cl pH 8.0, and EDTA). The strip wells were incubated at room temperature for 60 min, and then washed 4 times with phosphate buffered saline (PBS: Na_2_HPO_4_, NaH_2_PO_4_, and NaCl) and 0.1% Triton X-100. Then, 50 µL of diluted Strep-HRP (blocking solution) was added to each well, and the strips were further incubated at room temperature for 60 min. After washing the wells 4 times each with PBS and with 0.1% Triton X-100, they were mixed with 50 µL of TACS-Sapphire™ colorimetric substrate, and allowed to stand in the dark for 10-15 min. The intensity of the blue color that developed in each well was read on a microplate reader set at 630 nm. After stopping the reaction by adding 50 µL of 5% phosphoric acid to each well, the absorbance of the yellow color was measured at 450 nm. Parallel experiments were conducted by substituting the test solution with an equivalent volume of dimethyl sulfoxide or distilled water to verify the effect of the vehicles on the enzyme activity. All the samples were tested in triplicate.

To determine the IC_50_ value for the inhibitors, the compounds were further tested using 5 different concentrations ranging from 5 µM to 500 µM and the average absorbance of each inhibitor concentration was plotted against the log of the concentration of inhibitor producing each absorbance value (semi-log plot) and the IC_50_ value for each plot was obtained using Regression Wizard from Sigma Plot version 10.0 (Systat, San Jose, CA). All the assays were conducted in two separate occasions, each time in triplicate. The results of these studies are presented as mean ± standard deviation (SD) and are reported in μM.

### Molecular modeling studies

Molecular docking computations were carried out on a Dell Precision 470n workstation with the RHEL 4.0 operating system using Glide 5.0. 3D Structures of the various test compounds were constructed using the fragment dictionary of Maestro 9.0. The geometry of the ligands was optimized by Macromodel program v9.5 using the Optimized Potentials for Liquid Simulations-all atom (OPLS-AA) force field [25] with the steepest descent followed by truncated Newton conjugate gradient protocol. The X-ray crystallographic structure of PARP-1 in complex with quinazolinedione, a PARP inhibitor (PDB ID: 1UK1) [26], obtained from the RCSB Protein Data Bank (PDB),was used to model the protein structure in this study. The protein was optimized for docking using the “Protein Preparation Wizard” and “Prime-Refinement Utility” of Maestro 9.0. The inhibitors were glide docked into the PARP-1 active site by the extra precision Glide docking method and upon completion of each docking calculation, 100 poses at most per ligand were allowed to generate. Identical binding poses with better Glidescore of each of the target compounds (where a lower value reflects a stronger binding than a higher one) were selected for use in determining the residues on the PARP active site participating in the binding to the ligands under evaluation.

### Assay of plasma glucose

The content of glucose in a plasma sample was measured using a commercially available colorimetric assay kit (Procedure No. 510, Sigma Chemical Co., St. Louis, MO) which is based on the method of Rabbo and Terkildsen [27]. In the assay, an aliquot of plasma sample is mixed with an enzyme-color reagent solution consisting of glucose oxidase, peroxidase, and *ortho*-dianisidine, and the reaction is allowed to proceed at 37°C for 30 min. The absorbance of the colored solution is read on a spectrophotometer at 450 nm. The concentration of glucose in the sample was calculated by reference to a glucose standard solution provided by the manufacturer and treated in identical manner as the plasma sample. The result was expressed in mg/dl of plasma.

### Assay of plasma insulin

The quantity of insulin released into the plasma was measured using a commercially available solid phase two-site Insulin ELISA immunoassay kit and strips coated with insulin MAb (Catalog No. IS130D, Calbiotech, Inc., Spring Valley, CA). This assay is based on a direct sandwich technique in which two monoclonal antibodies (enzyme (HRP)-conjugated anti-insulin and anti-insulin antibody bound to a microtitration well) are directed against separate antigenic determinants on the insulin molecule. Following a washing step to remove any unbound enzyme labeled antibody, the bound HRP complex is detected by reaction with TMB to yield a colored product that can be read on an ELISA plate reader. The results were expressed as μIU/ml.

### Assay of plasma total cholesterol

The concentration of total cholesterol in a plasma sample was measured using a commercially available enzymatic-colorimetric assay kit (Liquizyme^®^ Cholesterol, Beacon Diagnostics Pvt. Ltd., Navsari, India). In the assay, 10 μl of plasma sample was mixed with 1.0 ml of enzyme-color reagent solution (consisting of cholesterol esterase, cholesterol oxidase, peroxidase, phenol and 4-aminoantipyrine), incubated at 37°C for 5 min, and the absorbance of the solution was read on a spectrophotometer at 505 nm against a blank preparation (the enzyme-color solution). A standard preparation of cholesterol, treated in identical manner as the plasma sample, was analyzed alongside and used as a reference for calculating the plasma cholesterol content, in mg/dl.

### Assay of plasma triglycerides

The concentration of triglycerides in a plasma sample was measured using a commercially available enzymatic-colorimetric assay kit (Liquizyme^®^ Triglycerides, Beacon Diagnostics Pvt. Ltd., Navsari, India). In the assay, 10 μl of plasma sample was mixed with 1.0 ml of enzyme-color reagent solution (consisting of lipase, glycerol kinase, Mg^++^, glycerol phosphate oxidase, and peroxidase), incubated at 37°C for 5 min, and the absorbance of the solution was read on a spectrophotometer at 500 nm against a blank preparation (the enzyme-color solution). A standard preparation of triglyceride, treated in identical manner as the plasma sample, was analyzed alongside and used as a reference for calculating the plasma triglyceride content, in mg/dl.

### Assay of plasma malondialdehyde

The formation of malondialdehyde (MDA) in the plasma was measured as TBARS by the method of Buege and Aust [28]. In the assay, 100 μl of plasma sample was mixed with 900 μl of a TCA-TBA-HCl reagent (containing 15% TCA, 0.375% TBA and 0.25 N HCl in distilled water). After incubation at 95°C for 60 min, cooling to room temperature, and centrifugation at 1000 x g for 10 min, the absorbance of the clear solution was read on a spectrophotometer at 535 nm against a blank preparation (the TCA-TBA-HCl reagent solution). The concentration of MDA in the plasma sample was calculated by reference to a calibration curve prepared from serial dilutions of a stock solution of TEP, treated in identical manner as the plasma sample, and was expressed in nM/ml.

### Assay of plasma GSH

The content of GSH in a plasma sample was measured using the method of Ellman [29] and in which this thiol compound is reacted with DTNB to form a colored product that is measured on a spectrophotometer. In the assay, a 100 μl aliquot of plasma sample was mixed with an equal volume of 5% metaphosphoric acid, and the mixture centrifuged at 2000 rpm for 5 min. A 100 μl portion of the supernatant was mixed with 1.9 ml of 0.1 M phosphate buffer pH 8.0 and 20 μl of 0.02 M DTNB, and the absorbance of the solution was read on a spectrophotometer at 412 nm against a blank preparation (prepared in identical manner as the plasma sample but lacking the plasma). The concentration of GSH in the plasma sample was calculated by reference to a calibration curve for GSH prepared from serial dilutions of a GSH stock solution that were treated identical manner as the plasma sample. The result was expressed as μM/ml.

### Assay of nitrite

Evidence of the formation of NO in the plasma was obtained indirectly by measuring the content of nitrite by the method of Kauser et al. [30] which is based on the use of the Griess reagent. In the assay, a 100 μl aliquot of plasma sample was mixed with 100 μl of 5% metaphosphoric acid, and centrifuged at 2000 rpm for 5 min. A 100 μl aliquot of the supernatant was mixed with 100 μl of Griess reagent (containing 0.1% NED 2HCl and 1% sulfanilamide in 5% phosphoric acid), and the absorbance of the solution was read on a spectrophotometer at 546 nm against a blank preparation (prepared in identical manner as the plasma sample but lacking the plasma). The concentration of nitrite in the sample was calculated by reference to a calibration curve prepared from serial dilutions of a stock solution of sodium nitrite that were treated in identical manner as the plasma sample. The result was expressed in nM/ml.

### Statistical analysis of the data

Results are expressed as the mean ± SEM for n = 6. Intergroup comparisons were carried out by Student’s t-test, one-way ANOVA, and Neuman-Keuls post hoc test. Differences were considered to be significant when p was <0.05.

## Results

### PARP-1 inhibitory action

3-AB, NIC and TAU were tested for their ability to inhibit PARP-1 using a colorimetric assay kit. The results of this test indicated that a wide difference in inhibitory potency existed among these compounds. In terms of their mean ± SD IC_50_ values, 3-AB (33 ± 1.8 μM) was about 6.4-fold more potent than NIC (210 ± 2.9 μM) and about 9.7-fold more potent than TAU (320 ± 3.4 μM).

### Molecular docking experiments on the active site of PARP-1

**Figure 1:**
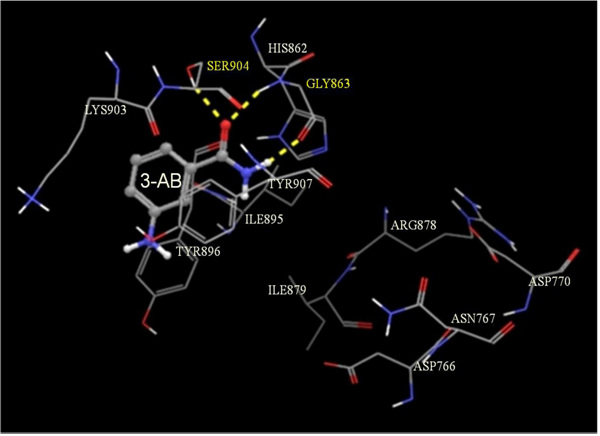
**Glide-predicted binding pose for 3-AB within the active site of PARP-1.** The inhibitor is shown in ball and stick model while the active site amino acids are shown in stick model. The yellow dotted lines indicate probable H-bonds between the inhibitor and the active-site amino acids. Amino acids participating in the H-bonding are shown in yellow.

**Figure 2:**
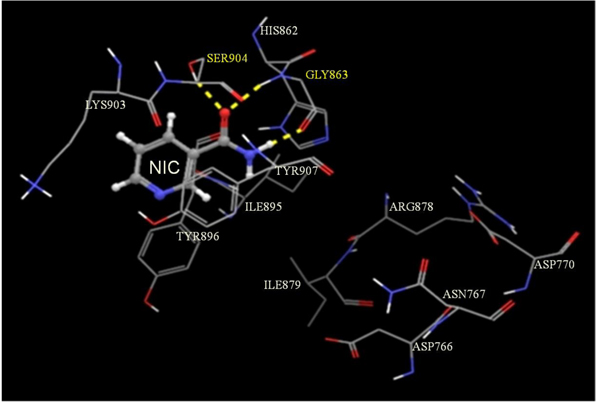
**Glide-predicted binding pose for NIC within the active site of PARP-1.** The inhibitor is shown in ball and stick model while the active site amino acids are shown in stick model. The yellow dotted lines indicate probable H-bonds between the inhibitor and the active-site amino acids. Amino acids participating in H-bonding are shown in yellow.

**Figure 3:**
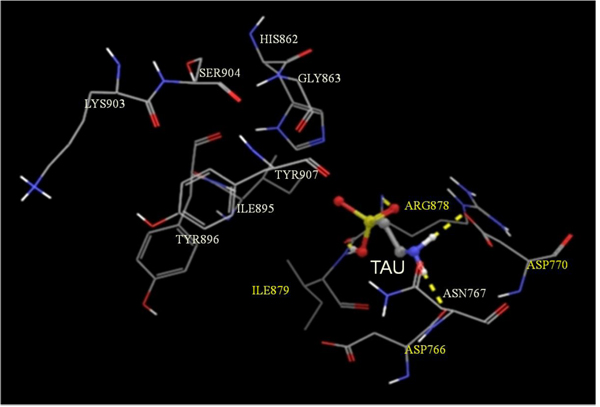
**Glide-predicted binding pose for TAU within the active site of PARP-1.** The inhibitor is shown in ball and stick model while the active site amino acids are shown in stick model. The yellow dotted lines indicate probable H-bonds between the inhibitor and the active-site amino acids. Amino acids participating in H-bonding are shown in yellow.

PARP-1 is a polypeptide whose activation requires binding as a homodimeric protein to a nicked DNA. This polypeptide possesses a highly conserved organization consisting of three main domains: a N-terminal DNA-binding domain which acts a DNA nick sensor, a central portion designated as the automodification domain (AMD) and which contain regions for dimerization and for modulating interactions with DNA and with proteins, and a C-terminal region, representing the most conserved part of the enzyme, and capable of catalyzing poly(ADP-ribose) synthesis and of binding to target proteins [31]. In turn, this active site, also known as cat PARP, can be divided into an acceptor (adenosine) site and a donor (NIC) site. The acceptor site is occupied by the ADP moiety of poly(ADP-ribose) and the donor by NAD^+^. In the donor site, three subsites are described: a NIC-ribose (NI) binding site, a phosphate (PH) binding site and an adenine-ribose (AD) site [31]. To be able to shed some light on the molecular mechanism of action of these inhibitors, we performed docking studies of these three inhibitors at the active site of PARP-1. As shown in Figures 1, 2, 3, molecular docking of 3-AB (Figure 1), NIC (Figure 2) and TAU (Figure 3) on the active site of PARP-1 reveals that while the amide group of 3-AB and NIC are seen hydrogen bonding to the hydroxyl group of Ser^904^ and amino and carbonyl groups of the backbone of Gly^863^ at the NI binding site of PARP-1, the highly polar TAU entered into hydrogen bonding at the AD site with the carbonyl group on the backbone of Asp^766^ and the carboxyl group of Asp^770^ via its β-amino group and to the carbonyl and amino groups on the backbone of Arg^878^ via its sulfonic acid group. Assessment of ligand binding efficiency based on docking scores indicated a good correlation with the PARP-1 inhibitory actions of the test compounds (-8.38 for 3-AB; -7.40 for NIC; -4.30 for TAU).

### Effects of STZ and treatment compounds on the plasma glucose and insulin levels

**Figure 4:**
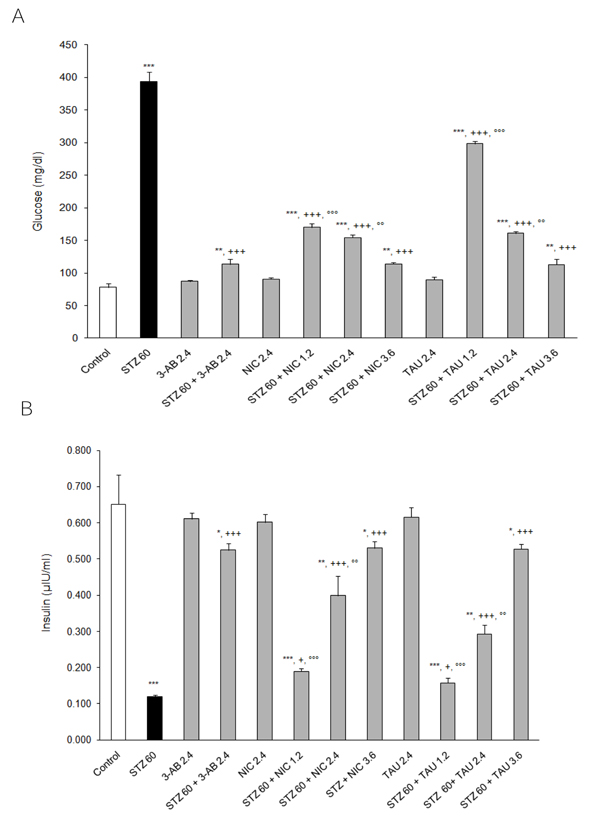
**Effects of 3-AB, NIC and TAU on plasma (A) glucose and (B) insulin of diabetic rats.** Data are presented as mean ± S.E.M. for n = 6. Statistical comparisons were significantly different at **P<0.01 and ***P<0.001 vs. Control; at ^++^P<0.01 and ^+++^P<0.001 vs. STZ; and at °P<0.05, °°P<0.01 and °°°P<0.001 vs. 3-AB.

From the data presented in Figure 4A it can be seen that while STZ caused a massive increase in plasma glucose (~404%, p<0.001 vs. control), a pretreatment with 2.4 mM/kg of 3-AB was able to attenuate this increase significantly (only 45% increase, p<0.001 vs. STZ) and to a greater extent than that byn equimolar dose of either NIC (~98% increase, p<0.001 vs. STZ) or TAU (106% increase, p<0.001 vs. STZ). At a dose of 1.2 mM/kg, NIC was more effective than TAU (increases equal to 117% and 282% above control, respectively; with p<0.01 and p<0.001 vs. STZ, respectively), but when the dose of both compounds was increased to 3.6 mM/kg, their attenuating effect on the plasma glucose was not only equal with each other but also equal to the action derived from a 2.4 mm/kg dose of 3-AB.

While a 60 mg/kg i.p. dose of STZ induced type 2 diabetes in the rat and suppressed the insulin output by about 82% of the control value (p<0.001), a pretreatment with any of the test compounds was able to preserve the ability of pancreas to secrete insulin following a treatment with STZ (Figure 4B). In parallel with the results seen for the plasma glucose, at 2.4 mM/kg, 3-AB was more effective than equimolar doses of either NIC or TAU (decreases of 21%, p<0.05, 40% p<0.01, and 56%, p<0.001, respectively). Increasing the dose of TAU and NIC to 3.6 mM/kg led to protective actions than were of equal magnitude (only 20% decrease, p<0.05 vs. control) and greater than that achieved with either compound at 2.4 mM/kg (Figure 4B).

### Effects of STZ and treatment compounds on plasma lipids

**Figure 5:**
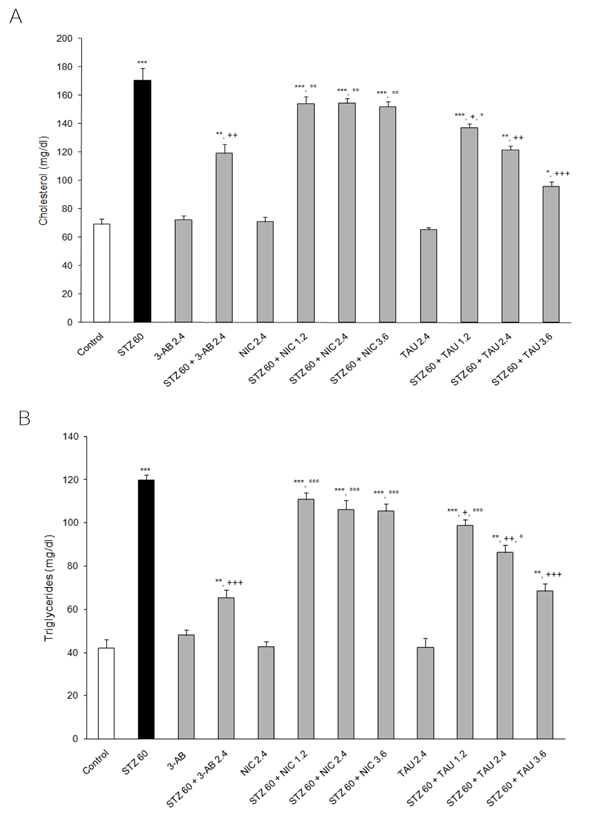
**Effects of 3-AB, NIC and TAU on plasma (A) total cholesterol and (B) triglycerides of diabetic rats.** Data are presented as mean ± S.E.M. for n = 6. Statistical comparisons were significantly different at **P<0.01 and ***P<0.001 vs. Control; at ^+^P<0.05, ^++^P<0.01 and ^+++^P<0.001 vs. STZ; and at °P<0.05, °°P<0.01 and °°°P<0.001 vs. 3-AB.

As shown in Figure 5A, STZ elevated the plasma total cholesterol by 147% over the control value (p<0.001). The same Figure also indicates that a pretreatment with 2.4 mM/kg dose of either 3-AB (only 72% increase, p<0.01 vs. STZ) or TAU (only 76% increase, p<0.01 vs. STZ) but not one with NIC (123% increase, p<0.001 vs. control). While at a 1.2 mM/kg dose TAU exerted a marginal attenuating effect (98% increase, p<0.001 vs. control), at this dose NIC produced an effect that was identical to that achieved at twice the dose. Raising the dose of a treatment compound to 3.6 mM/kg led to a marked enhancement of the anticholesterolemic action of TAU (only 39% increase, p<0.001 vs. STZ) but had no effect on the potency of NIC (120% increase, p<0.001 vs. control).

From the results shown in Figure 5B, it is apparent that STZ raised the plasma triglyceride level to a significant extent relative to the control value (184%, p<0.001). The same Figure also indicates that a pretreatment with 2.4 mM/kg dose of either 3-AB (only 55% increase, p<0.001 vs. STZ) or TAU (105% increase, P<0.01 vs. STZ), but not one with NIC (152% increase) to SZT was able to attenuate the effect of STZ. Raising the dose of the pretreatment compound to 3.6 mM/kg enhanced the attenuating action of TAU (only 62% increase, p<0.001 vs. STZ.) but not that of NIC (152% increase) on the plasma triglycerides. At 1.2 mM/kg, TAU had a negligible counteracting effect on the action of STZ on the plasma triglyceride level (~135% increase).

### Effects on indices of oxidative stress

**Figure 6:**
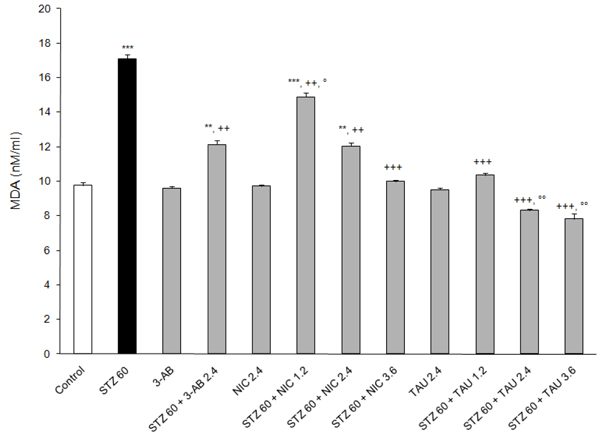
**Effects of 3-AB, NIC and TAU on plasma MDA of diabetic rats.** Data are presented as mean ± S.E.M. for n = 6. Statistical comparisons were significantly different at *P<0.05, **P<0.01 and ***P<0.001 vs. Control; at ^++^P<0.01 and ^+++^P<0.001 vs. STZ; and at °P<0.05 and °°P<0.01 vs. 3-AB.

**Figure 7:**
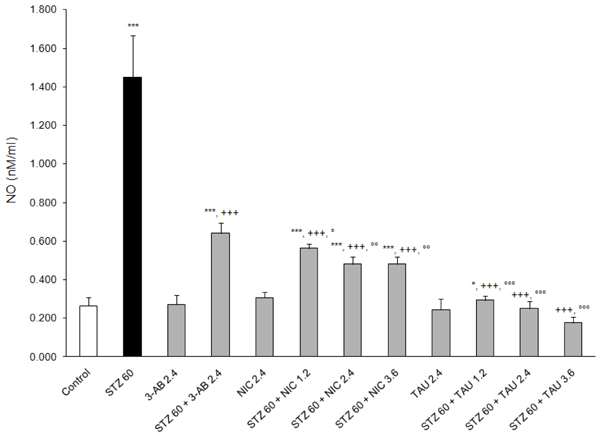
**Effects of 3-AB, NIC and TAU on plasma NO of diabetic rats.** Data are presented as mean S.E.M. for n = 6. Statistical comparisons were significantly different at *P<0.05 and ***P<0.001 vs. Control; at ^+++^P<0.001 vs. STZ; and at °P<0.05, °°P<0.01 and °°°P<0.001 vs. 3-AB.

**Figure 8:**
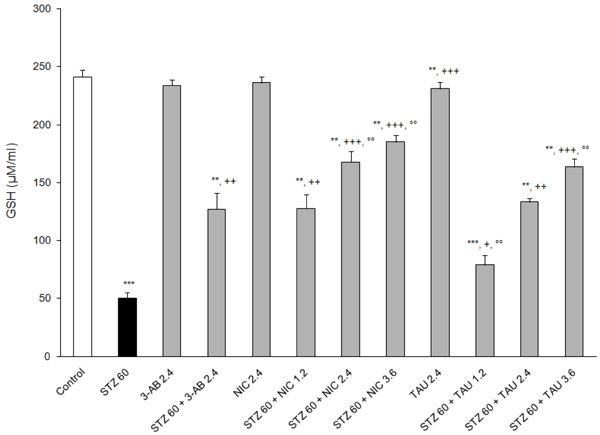
**Effects of 3-AB, NIC and TAU on plasma GSH of diabetic rats.** Data are presented as mean ± S.E.M. for n = 6. Statistical comparisons were significantly different at **P<0.01 and ***P<0.001 vs. Control; at ^+^P<0.05, ^++^P<0.01 and ^+++^P<0.001 vs. STZ; and at °°P<0.01 vs. 3-AB.

The occurrence of oxidative and nitrosative stress as a result of STZ-induced diabetes was assessed on the basis of the plasma MDA (Figure 6), NO (Figure 7) and GSH (Figure 8) levels. By itself, SZT increased the plasma levels of MDA (by75%) and of NO (by ~450%) but lowered that of GSH (by 70%) to a significant extent (p<0.001 vs. corresponding control values) (Figure 6). A pretreatment with any of the test compound resulted in a significant attenuation of the oxidative stress induced by STZ. Thus, in terms of the MDA levels, although all the test compounds demonstrated a significant counteracting effect on the formation of this end product of LPO, TAU was the most effective at all doses examined (6% increase at 1.2 mM/kg, ~15% below control at ≥2.4 mM/kg) levels (Figure 6). Likewise, the attenuating action of NIC was also dose-related, with the increases in MDA formation ranging from 2-52% above control. On the other hand, at a dose of 2.4 mM/kg 3-AB and NIC were equipotent in lowering MDA formation (~24% increase above control, p<0.05).

NO is one of the metabolic products of STZ responsible for DNA strand breakage in pancreatic cells and for the formation of the highly toxic peroxynitrite anion following interaction with reactive oxygen species (ROS) [32]. In this study, STZ was found to cause a marked increase in plasma NO (by ~450%, p<0.001 vs. control), an effect that was lessened to different extents by a 2.4 mM/kg dose of 3-AB (143% increase), NIC (83% increase) and TAU (5% decrease) relative to the control value and which differed significantly from the value with STZ alone (p<0.001) (Figure 7). Varying the dose of TAU between 1.2 and 3.6 mM/kg had a very a small dose-related increasing effect on the attenuating action of both TAU and NIC on NO formation (Figure 7).

GSH is a biomolecule that functions as an antioxidant by participating in the detoxification of electrophilic/oxidizing xenobiotics, free radicals and peroxides [33]. During situations of oxidative stress, its intracellular stores may become depleted by conjugating reactions, by binding to susceptible cysteine residues in macromolecules to form mixed disulfides, and by oxidation to its disulfide form [33]. Hence its levels in tissues and in the circulation may reflect the extent of the oxidative state. In the present study, an acute treatment with STZ caused a drastic (70%) reduction of the circulating GSH (Figure 8). The administration of 3-AB, NIC or TAU as a pretreatment to STZ led to significant protection against the loss of GSH. Thus, at a dose of 2.4 mM/kg, NIC (40% decrease) was somewhat better than 3-AB (45% decrease) or TAU (48% decrease) in preserving the levels of plasma GSH. While NIC was more protective than TAU at doses ≤ 2.4 mM/kg, they provided equal protection at a dose of 3.6 mM/kg (~33% decrease) (Figure 8).

## Discussion

In the present study, diabetes was induced by the intraperitoneal administration of STZ. This methylnitrosoureido derivative of D-glucose is well suited as pharmacological tool for studying the effects of compounds with PARP-1 inhibitory action and with potential for preventing the onset of type 1 diabetes in experimental animals. Following its entry into pancreatic β-cells, STZ will be toxic to cells because it depresses the levels of NAD^+^ and ATP as a result of several possible mechanisms. First, metabolism of its nitrosoureido moiety can yield chemical species (methyl carbonium ion, methyl radical) for alkylating DNA, inhibiting the incorporation of precursors into DNA, and causing DNA strand breaks [34,35]. There also evidence to indicate that STZ can enhanced the production of NO, peroxynitrite and superoxide radical for widespread DNA strand breakage [9,36-38]. Hence, and irrespective of what causes DNA breakage, protracted activation of PARP-1 to repair the damage and ensure pancreatic cell survival will lead to β-cell dysfunction and culminate in death by necrosis or apoptosis [9,37]. Additional factors that contribute to the development of diabetes by STZ are the decrease in the pancreatic GSH level with a corresponding increase in GSSG [35,39], and the inhibitory action of the diabetogenic agent on the activity of islet O-GlcNAcase, an enzyme that cleaves terminal β-*O*-GlcNAc residues from modified nucleocytoplasmic proteins acting as a glucose sensor [40,42].

The three compounds evaluated in the present study are endowed with biological properties that are of benefit in preventing or delaying the onset of type 1 diabetes and to minimize the development of complications of diabetes. In this context, while 3-AB and NIC have been extensively studied for their PARP-1 inhibitory actions, in the case of TAU such link appears lacking. Although both 3-AB and NIC are examples of PARP inhibitors that are presently considered to be of low potency when compared to compounds designated as second- and third-generation inhibitors, they are still used experimentally to explore the function of the PARP family of enzymes [42]. Among PARP inhibitors, NIC remains a subject of clinical interest because of its proven antidiabetogenic effects on an experimental level, ready availability, wide therapeutic index, and infrequent reports of adverse effects when used in high doses on a chronic basis [43]. More importantly, its lower potency in comparison with 3-AB or newer benzamides guarantees that some PARP activity will be left for the cell to carry out normal DNA repairs [43].

In vitro testing of 3-AB, NIC and TAU for their PARP-1 inhibitory activity indicated that 3-AB was about 6.4-fold more potent than NIC and about 9.7-fold more potent than TAU. The experimental IC_50_ values for both 3-AB and NIC fall within the range of values reported for these compounds in the scientific literature [43,44]. With the help of molecular docking studies on an X-ray crystallographic structure of PARP-1 in complex with quinazolinedione, a PARP inhibitor, it was possible to establish the binding characteristics of each of the test compounds at the active site of PARP. While the more potent PARP inhibitors, 3-AB and NIC, were found to enter into hydrogen bond associations with Ser^904^ and Gly^863^ at the NI binding site of the active site, the much weaker inhibitor, TAU, was forming hydrogen bonds with Asp^766^, Asp^770^ and Arg^878^ at the AD site of the active site. Calculation of the corresponding docking scores for each compound, which are inversely related to the binding affinities, suggested 3-AB to be more tightly bound than NIC to the active site of the enzyme than NIC, and TAU to be weakly bound to PARP-1. In common with most PARP inhibitors, the three compounds under evaluation are inhibiting the enzyme in a competitive manner since they block the binding of NAD^+^ to the catalytic domain of PARP-1 [44].

In addition to differences in inhibitory activity imposed by their structural features [44], the action of 3-AB and NIC as inhibitors of PARP-1 inhibitors is also found to depend on the time of their administration following a treatment with STZ. Indeed, a study in rats evaluating 3-AB alongside NIC for their protective actions against STZ-induced β-cell toxicity, verified that protection by 3-AB remained when this compound was given up to 120 min after STZ, the optimum being 45 min, but that it completely disappeared when given 240 min after STZ. In contrast, protection by NIC required an administration earlier than 120 min. In addition to normalizing the blood glucose and preserving the plasma insulin levels, 3-AB was found to limit the decrease in pancreatic insulin content in a dose-dependent manner and to a greater extent than NIC when given at the same mg/kg (75, 150, 300) doses [15]. Moreover, in the same study it was verified that islet cells incubated in the presence of 3-AB or NIC were able to retain their ability to secrete insulin in response to a challenge with glucose, with the potency being greater if the compound was added early in the incubation since it decreased as function of the time of addition after STZ.

A role of PARP activation in pancreatic islet cell damage by STZ can be ascertained from the circulating levels of both insulin and glucose, with the type of diabetes being a determined by the dose of STZ administered [45]. In the present study, a 60 mg/kg dose of STZ was found to induce type 2 diabetes since the levels of immunoreactive insulin were still measurable at 24 hr after STZ. To preclude the loss in inhibitory activity observed when a PARP-1 inhibitor is given as a posttreatment to STZ, all the treatment compounds were administered 45 before STZ. When given at 2.4 mM/kg dose prior to STZ, 3-ABC, NIC and TAU were able to counteract the deleterious effect of STZ on the pancreas as determined from the increases in plasma insulin levels relative to STZ alone, and which were directly proportional to their PARP-1-inhibitory potency. The effect of both NIC and TAU in preserving the insulin-secreting ability of the pancreas was enhanced by raising their doses to 3.6 mM/kg, with TAU then becoming equipotent to NIC. In parallel with the results for plasma insulin, all the test compounds were found to reduce the hyperglycemic response induced by STZ when administered at a 2.4 mM/kg dose, with the effect being directly proportional to the PARP-1 inhibitory activity. At 1.2 mM/kg NIC was more potent than TAU in reducing the plasma glucose due to STZ; but this difference was not observed when the two compounds were administered at a dose of 3.6 mM/kg. Surprisingly, at this particular dose the effects of TAU and NIC on the plasma glucose became equal to that of a 2.4 mM/kg dose of 3-AB.

In rodents, the induction of diabetes with a STZ is known to lead to a hyperlipidemia characterized by elevations in the circulating levels of total cholesterol, triglycerides, very low density lipoprotein and low density lipoprotein-cholesterol and by a decrease of high density lipoprotein-cholesterol [36,39,47]. In the present study, diabetes by STZ led to a marked increase in both the plasma cholesterol and plasma triglyceride levels at 24 hr after STZ. At a 2.4 mM/kg dose, 3-AB and TAU, but not NIC, were able to attenuate the elevations in plasma lipids induced by STZ to a significant extent. In this instance, both compounds attenuated the cholesterol level to about the same extent and 3-AB was more potent than TAU in lowering the triglycerides level seen with STZ. Also, while a treatment with TAU at doses ranging from 1.2 to 3.6 mM/kg produced a dose-related decreasing effect on both plasma lipids, one with NIC at the same doses had a negligible effect. These results clearly suggest that the actions of 3-AB, NIC and TAU in general and of NIC in particular are more dependent on their intrinsic pharmacological actions than on their PARP-1 inhibitory potencies.

Oxidative stress is a hallmark of STZ-induced diabetes since this diabetogen can promote the formation of ROS and NO in the pancreas [35,38,48] and other major organs [49]. In β-cells, ROS and NO produced from the metabolism of STZ itself and from other sources, especially from the oxidation of glycated proteins [50], can attack islet cell membranes to cause oxidative damage and promote the release lipid peroxidation products such as MDA and conjugated dienes [23]. This process is compounded by the subsequent oxidative modifications to membranes lipids to generate new free radicals, reactive aldehydes and lipid peroxides, especially hydrogen peroxide, which are thought to play an important role in the development of diabetes and in the pathogenesis of diabetic complications since they can damage DNA and other cell components [7,49,51]. Pancreatic β-cells are quite susceptible to the effects of peroxides because of their limited contents of catalase and peroxidase [7] and because STZ also lowers the activities of antioxidant enzymes such as glutathione peroxidase [52]. STZ-mediated pancreatic β-cell damage may also be the result of STZ attacking mitochondria to impair ATP generation and increase ADP, thus providing mitochondrial xanthine oxidase with a substrate for the formation of uric acid and superoxide anion radicals. Alternatively, STZ can directly activate pancreatic xanthine oxidase to enhance superoxide anion radical formation. In turn, exposure of superoxide anion radicals to STZ can generate hydroxyl radicals [53].

The present study finds that all the test compounds were able to lower the plasma levels of NO and MDA, a secondary product of LPO, and to increase the plasma GSH content relative to plasma levels from animals on STZ alone. In all likelihood, these changes reflect the intracellular status of those organs and tissues, particularly erythrocytes, being affected by STZ-induced diabetes along with the activities of those intracellular systems responsible for their formation [54], Furthermore, at a dose of 2.4 mM/kg TAU was the most potent and 3-AB the least potent in attenuating MDA and NO formation, and NIC was the most potent and 3-AB the least potent in preventing the loss of GSH as a result of a post-treatment with STZ. Without exceptions, the protective effects of TAU and NIC were dose-related.

The reduction in LPO by these compounds is not unexpected since they have all shown antioxidant properties in vivo, in vitro and ex vivo. Thus, 3-AB was found to ameliorate peroxynitrite-induced cytotoxicity [55], to effectively scavenge the hydroxyl radical and to protect proteins against oxidation, but without been able to prevent FeCl_2_-induced LPO [56]. NIC can contribute to the maintenance of the intracellular GSH since it is a substrate for the de novo synthesis of NAD^+^. In turn, NAD^+^ undergoes metabolic conversion to NADP, which in its reduced form is an obligatory cofactor for the redox cycling of GSH with its disulfide form GSSG under the mediation of glutathione reductase [57]. Furthermore, since GSH is a cosubstrate for glutathione peroxidase, it will play a protective role against ROS generated during the oxidative metabolism of glycated proteins formed as a result of chronic hyperglycemia [54,57]. In rats, feeding a diet supplemented with NIC for 4 weeks prior to a treatment with STZ (40 mg/kg, i.v.) resulted in lower pancreatic TBARS values than in animals consuming an unsupplemented or NIC-deficient diet but without impacting on the liver MDA values [58]. The same study found the liver contents of GSH and vitamin E to be higher in animals on the NIC-supplemented diet than in animals on the unsupplemented and NIC-deficient diets. Furthermore, in an in vitro study in which a rat brain was subjected to cerebral ischemia and/or reperfusion, NIC was shown to reduce neuronal ROS production, Ca^++^ influx, apoptosis and cell injury in a dose-related manner [59].

In addition to its protective actions against STZ-induced hyperglycemia and hypoinsulinemia [20], TAU is reported to prevent glucose-induced membrane LPO in cultured rat mesangial cells [60] and in the liver and kidney of alloxan-treated rats [61] as inferred from the levels of MDA, hydroperoxides and conjugated dienes content; to attenuate the formation of chemiluminescent products of LPO when egg yolk phosphatidylcholine liposomes were incubated with 2,2’-azo-bis-(2-amidino-propane) hydrochloride [AAPH], a water soluble oxygen radical generator, possibly because of its sulfonate group [62]; and to prevent hemolysis, but not LPO, when intact canine erythrocytes were exposed to AAPH [63]. In mice, supplementation of the drinking water with TAU reduced myocardial oxidative stress due to iron overload and reversed the changes in MDA, hexanal, 4-hydroxynonenal, GSH and GSSG production, and elevated the GSH/GSSG ratio [64]. Feeding rats with TAU (0.1%) as part of the drinking water was shown to protect the myocardium against free radical damage as a result of in vitro electrolysis-induced ischemia/reperfusion injury but without the ability to sequester superoxide anion radicals [65]. However, the apparent lack of correlation between the beneficial antioxidant effects manifested by TAU in diabetes and other pathological conditions, and the inability of this sulfur compound to directly scavenge free radicals (e.g., hydroxyl, superoxide anion, peroxynitrite) or to react with H_2_O_2 _[63,66], has cast doubts on an antioxidant action as the mechanism whereby TAU protects the pancreas and other major organs against injury by oxidative stress However, a recent report has attempted to provide a unifying view of this controversial issue [67]. In brief, it is hypothesized that in diabetes the formation of ROS in the mitochondria of pancreatic islet cells exposed to high glucose levels will negatively impact of the levels of pancreatic mitochondrial TAU. Since TAU is required for the formation of mitochondrial TAU-conjugated tRNAs which mediate the normal translation of mitochondrial-encoded proteins, including protein components of the respiratory chain, a deficiency of TAU will impair the flow of electrons through the respiratory chain, promote the accumulation of electron donors, reduce ATP production, and divert electrons from the respiratory chain to oxygen to generate superoxide anion. Hence, replenishment of the mitochondrial stores of TAU will restore respiratory chain function and ATP synthesis at the cost of superoxide anion formation [67], and, more importantly, spare pancreatic cells from death.

## Conclusions

The results of the present work indicate that TAU possesses PARP-1 inhibitory activity and that this activity is rather weak when compared to classical PARP-1 inhibitors such as 3-AB and NIC. Through molecular docking analysis, TAU is found to bind to subsite of the active site of PARP-1 that is different from that shared by 3-AB and NIC. When administered to rats at a 2.4 mM/kg dose 45 min before a 60 mg/kg dose of STZ, 3-AB, NIC and TAU demonstrated common attenuating actions on the hyperglycemia, hypoinsulinemia and oxidative stress induced by STZ, with 3-AB exhibiting the greatest potency and TAU the least. However, at a dose of 3.6 mM/kg the effects of TAU and NIC against STZ-related alterations became either equal or greater than those by a 2.4 mM/kg dose of 3-AB. Irrespective of the dose used, TAU was the most protective among the compounds tested against oxidative stress by STZ. On the other hand, the lack of effect of NIC, a more potent PARP-1 inhibitor than TAU on hyperlipidemia by STZ suggests that PARP-1 inhibitory potency is not a reliable predictor of the type and extent of the effect that a PARP inhibitor may have on the plasma lipids associated with diabetic hyperlipidemia.

## Abbreviations

TMB: 3,3´,5,5´-tetramethylbenzidine; TBARS: thiobarbituric acid reactive substances; TCA: trichloroacetic acid; TBA: thiobarbituric acid; HCl: hydrochloric acid; TEP: 1,1,3,3-tetraethoxypropane; GSH: reduced glutathione; GSSG: oxidized glutathinoe; DTNB: 5,5’-dithiobis(2-nitrobenzoic acid); NED 2HCl: N-1-naphthylethylenediamine hydrochloride.

## Competing interests

The authors declare that they have no competing interests.

## Authors’ contributions

KGP carried out all experimental work on live animals, performed all the biochemical assays and statistical analyses, helped with the collection of literature information, prepared the figures, and made editorial comments to the article. MRP performed the in vitro tests for PARP-1 inhibition, carried out the molecular modeling studies, prepared the figures for the molecular modeling results, and made editorial comments to the article. CLP conceived the project and guided its development, assembled, organized and interpreted the experimental data, and reviewed the pertinent scientific literature.
